# Fruit fly species (Diptera: Tephritidae) associated with fruit orchards in the province of Luya, Amazonas, Peru

**DOI:** 10.3389/finsc.2026.1789891

**Published:** 2026-03-18

**Authors:** Enistein Reyna-Rivera, Vilma Aguilar-Rafael, Wagner Meza-Maicelo, Henry W. Santillan-Culquimboz, Duber Banda-Martinez, Luis Cubas-Vasquez, Santos Leiva-Espinoza

**Affiliations:** 1 Escuela Profesional de Ingeniería Agrónoma, Facultad de Ingeniería y Ciencias Agrarias (FICA), Universidad Nacional Toribio Rodríguez de Mendoza de Amazonas (UNTRM), Chachapoyas, Peru; 2 Instituto de Investigación para el Desarrollo Sustentable de Ceja de Selva (INDES-CES), Universidad Nacional Toribio Rodríguez de Mendoza de Amazonas (UNTRM), Chachapoyas, Peru

**Keywords:** anastrepha, ceratitis, mitochondrial COI, morphology, trapping

## Abstract

In the province of Luya, the presence of fruit flies (Tephritidae) in fruit orchards causes significant losses to the family economy. This study aimed to identify, using morphological and molecular methods, the Tephritidae species associated with fruit hosts and to evaluate host preference. Fruits were collected from orchards at different geographic points in the province of Luya; these fruits were transferred to the laboratory under controlled conditions and placed in plastic containers on sterilized soil to favor pupation. When adults emerged, they were fed with sucrose. For morphological identification, adult females were used and pictorial keys were employed, while for molecular analysis, the cytochrome c oxidase subunit I (Cox1) region was amplified using the primers LCO1490/HCO2198 and LepF1/LepR1. After identification, fruit fly preference for specific fruit species was evaluated by associating the identified species with the fruits from which they emerged. Nine fruit fly species were identified, corresponding to eight species of the genus *Anastrepha* (*Anastrepha fraterculus*, *A. obliqua*, *A. striata*, *A. distincta*, *A. grandis*, *A. ornata*, *A. leptozona*, and *A. nolazcoae*) and one species of the genus *Ceratitis* (*Ceratitis capitata*), associated with 19 hosts from 11 botanical families. The fruits with the highest preference were *Psidium guajava* and *Campomanesia liniatifolia*. These results highlight the richness of *Tephritidae* in the province of Luya and represent the first records of these nine species for the Amazonas region, expanding their geographical distribution in Peru, emphasizing the utility of integrative approaches for reliable taxonomic identification.

## 1 Introduction

Fruit flies (Diptera: Tephritidae) represent one of the most important pest groups worldwide in fruit and horticultural systems (1). The Tephritidae family contains more than 5,000 species distributed across over 500 genera, with a wide presence in tropical and temperate regions (2, 3) of which approximately 40% are frugivorous species (4). These species cause damage during oviposition and the larval phase, as the internal feeding on the pulp induces premature ripening and early fruit drop, leading to considerable economic losses (5–7).

Sexual reproduction in *Tephritidae* is crucial for oviposition and larval development, as females, after copulation, deposit their eggs in the fruit, which enables the pest’s reproductive cycle (8). Studying sexual behavior and reproductive processes is essential for understanding pest species and developing more sustainable and environmentally friendly control methods (9). Furthermore, understanding these processes is key to designing control strategies, such as the release of sterile males, whose efficacy depends on the population fluctuation of females and males (10).

From a taxonomic perspective, the species of greatest economic importance are primarily concentrated in the genera *Anastrepha* Schiner, *Bactrocera* Macquart, *Ceratitis* MacLeay, *Dacus* Fabricius, *Rhagoletis* Loew, and *Toxotrypana* Gerstaecker (11). Among them, the genera *Anastrepha* and *Ceratitis* stand out in the Americas for their wide distribution, high adaptability, and severe impact on fruit production (12, 13).

The genus *Anastrepha* is endemic to the neotropical region and constitutes the most important group of fruit flies in the Americas, housing 328 recognized species in 28 groups, with a distribution extending from the southern United States to northern Argentina (14–17).

On the other hand, the genus *Ceratitis* is native to the afrotropical region and was introduced to the Americas in the early 20th century, with its first record in Brazil in 1905 (18–20). Since then, *Ceratitis capitata* has become one of the main fruit pests on the continent, due to its wide distribution, which includes over 260 species of host plants, facilitating its rapid spread and adaptation to various agricultural systems (21, 22).

In Peru, fruit farming is a strategic activity for the national economy, driven by the growth of agroexports; however, fruit flies represent one of the main phytosanitary threats, causing significant economic losses in various producing regions of the country (23). In the province of Luya, Amazonas region, a wide diversity of native and introduced fruit trees are cultivated, mainly in small family orchards for self-consumption and local marketing.

In this context, the coexistence of multiple fruit hosts in the province of Luya could favor the establishment and persistence of Tephritidae species, in accordance with what has been described for heterogeneous landscapes (24). Furthermore, it is becoming a potential refuge against the pressure exerted by fruit fly eradication programs implemented in nearby regions, such as Lambayeque (25), which represents a risk of repopulation into intervened areas and could compromise the sustainability of these actions.

Traditionally, fruit fly identification has been based on morphological and phenotypic characters (26). However, this approach has limitations in the case of cryptic species, immature stages, or when diagnostic traits are not evident, and it can be time-consuming or even prone to errors, often leading to misidentification (27, 28). For this reason, molecular identification has become a complementary tool that helps resolve ambiguities and increase taxonomic accuracy (29). For example, when insect species exhibit high morphological similarity that hinders identification based solely on external characters, the use of the mitochondrial cytochrome c oxidase subunit I (Cox1) gene through DNA barcoding has proven effective for their discrimination (30), including a wide diversity of species within the family Tephritidae (31). In particular, the analysis of genetic distances based on Cox1 sequences allows for the evaluation of genetic divergence, both within a species (intraspecific) and between different species (interspecific), providing key information on the degree of genetic differentiation in both cases (32).

Additionally, phylogenetic analysis based on Cox1 gene sequences provides an independent framework to confirm species-level identification and to evaluate the evolutionary relationships of the analyzed specimens through comparison with reference sequences available in public databases (33). However, Barr et al. (34) demonstrated that, in Tephritidae, a DNA barcoding approach based exclusively on the mitochondrial Cox1 marker does not allow unequivocal identification of some species within the *fraterculus* group, indicating limited resolution for distinguishing closely related species.

In this context, the objective of this study was to identify the species of fruit flies associated with fruit species in the province of Luya, Amazonas region, using morphological and molecular tools, and to evaluate host preferences.

## 2 Materials and methods

### 2.1 Study area

**Figure 1:**
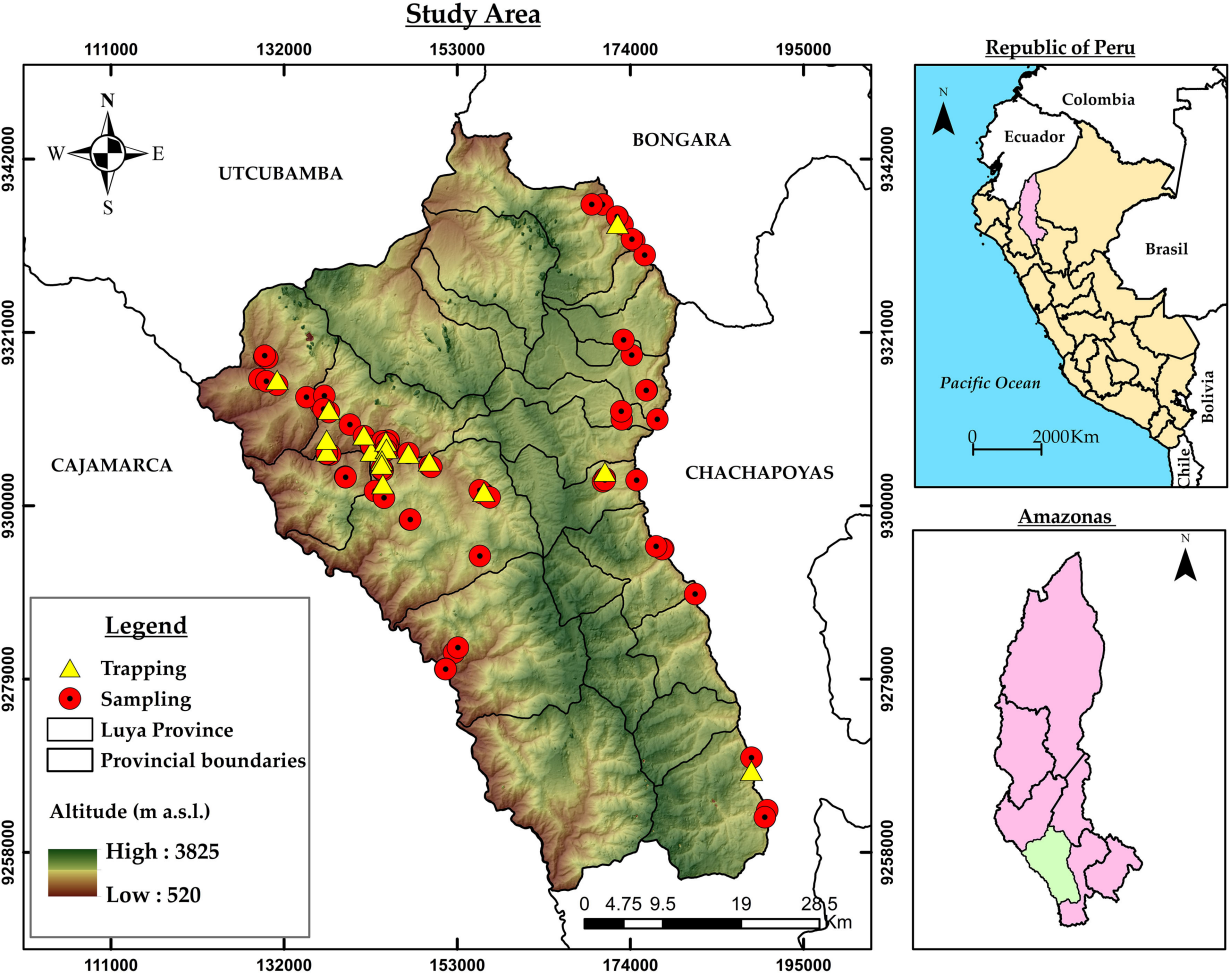
Geographical location of the study area (Amazonas, Peru) and spatial distribution of the sampling points (red points) and trapping points (yellow points) along the altitudinal gradient.

The study was conducted in the province of Luya, which is located in the southwestern part of the Amazonas region in Peru. The province covers approximately 3236.7 square kilometers and has an altitudinal gradient ranging from 520 to 3825 meters above sea level (**Figure 1**).

### 2.2 Trapping

Prior to collecting fruit, seventeen artisanal traps were installed in the fruit orchards of the province of Luya to confirm the presence of fruit flies (**Figure 1**; **Supplementary Table 1**). The traps were constructed from durable cardboard and yellow plastic sheets with an internal support for placing an adhesive sheet (Temocid). The traps were adapted to use hydrolyzed protein (CeraTrap^®^) (35) as a food attractant, which was placed in an internal dispenser to prevent direct contact with the adhesive surface. The traps were installed in representative orchards in the study area and suspended at an average height of 1.5 to 2.0 meters above the ground.

### 2.3 Fruit collection and fruit fly breeding

Sampling was conducted from August 2024 to June 2025 at 95 sites across the province of Luya (**Figure 1**), where 19 fruit species were identified. A non-probabilistic sampling method was employed to obtain infested fruits, selecting trees whose fruits exhibited visible symptoms of Tephritidae infestation, such as external spots, perforations, and tissue softening (36). At each sampling site, all fruits exhibiting symptoms were collected.

The fruits collected at each sampling site were transported in plastic containers of different sizes according to their size and were properly labeled. Then, they were taken to an environment conditioned for fruit fly rearing at the Centro de Promoción de la Investigación y Transferencia Tecnológica (PROCEPITT) at the National University of Toribio Rodríguez de Mendoza of Amazonas (UNTRM).

The fruit from each sampling site was disinfected with a 2% solution of sodium hypochlorite. To facilitate larval pupation, the fruit was placed in individual 30 cm × 20 cm × 10 cm containers with sterilized agricultural soil and then placed in an autoclave at 121 °C and 15 psi for one hour. The total weight of the fruit for each species was approximately 6 kg. Finally, we covered the containers with tulle fabric to prevent insects from entering or escaping.

Throughout the entire rearing period, the room was equipped with a heater to maintain a temperature between 25 and 28 °C and a humidifier to maintain relative humidity close to 80%. The photoperiod was set to a 12-hour light and 12-hour dark cycle. After emergence, the adults were fed a 30% sucrose solution provided on a piece of damp cotton placed on the tulle fabric surface. Once the fruit flies reached physiological and sexual maturity, the number of males and females was recorded.

### 2.4 Morphological identification

Five adult female fruit flies were selected from each sampling point for morphological identification. The specimens were taxonomically determined for diagnostic purposes by applying the morphological characters of the wing pattern and aculeus. This was done using a pictographic key constructed from these diagnostic characters. The identification was then supported using taxonomic keys and diagnostic descriptions from specialized literature (37–39). Technical references were also used for general fruit fly species identification (40).

Photographs of the adult females were obtained using a Stemi 508 stereomicroscope attached to an Axiocam 208 camera (Zeiss, Oberkochen, Germany). The specimens were euthanized beforehand in a cyanide lethal chamber (41), which enabled better handling and positioning for proper photodocumentation.

### 2.5 Molecular identification

#### 2.5.1 DNA extraction

DNA was extracted from the insect legs using the Quick-DNA™ Miniprep kit (Zymo Research), following the manufacturer’s instructions, due to its efficiency in obtaining high-quality total DNA from small amounts of animal tissue, as validated by the manufacturer (42) (Zymo Research, s.f) and previously reported in entomological studies (43). The reactions were prepared in a final volume of 25 μL, consisting of 12.5 μL of Master Mix (Promega, Madison, Wisconsin, USA), 1.25 μL of each primer at a concentration of 10 μM, 5 μL of nuclease-free water, and 5 μL of DNA.

#### 2.5.2 Amplification

The amplification of the mitochondrial COI gene was performed using conventional PCR with the primers LCO1490/HCO2198 and LepF1/LepR1 (**Table 1**). Each reaction was prepared in a final volume of 25 μL, including a negative control without DNA to rule out potential contamination. The primer pair LCO1490/HCO2198 successfully amplified all specimens, whereas LepF1/LepR1 amplified all specimens except *Anastrepha distincta*.

**Table 1:** Primer sequences used for the amplification of the mitochondrial COI gene, indicating their direction and methodological reference.

Primers	Sequence	Direction	Method
LCO1490	5’-GGTCAACAAATCATAAAGATATTGG-3’	Forward	Folmer et al. (44)
HCO2198	5’-TAAACTTCAGGGTGACCAAAAAATCA-3’	Reverse	
LepF1	5’-ATTCAACCAATCATAAAGATATTGG-3’	Forward	Hebert et al. (45)
LepR1	5’-TAAACTTCTGGATGTCCAAAAAATCA-3’	Reverse	

For the primer pair LCO1490/HCO2198, amplification was performed following the protocol described by Folmer et al. (44), which included an initial denaturation at 94 °C for 3 minutes, followed by 39 cycles of denaturation at 94 °C for 1 minute, hybridization at 52 °C for 1 minute, and extension at 72 °C for 1 minute, concluding with a final extension at 72 °C for 10 minutes. Additionally, for the primer pair LepF1/LepR1, the protocol proposed by Hebert et al. (45), was used, which consisted of an initial denaturation at 94 °C for 1 minute, followed by five cycles of denaturation at 94 °C for 30 seconds, hybridization at 45 °C for 40 seconds, and extension at 72 °C for 1 minute. Subsequently, 35 additional cycles were performed with denaturation at 94 °C for 30 seconds, hybridization at 55 °C for 40 seconds, and extension at 72 °C for 1 minute, finishing with a final extension at 72 °C for 10 minutes.

The verification of PCR products was carried out using 1% agarose gel electrophoresis, loading 1.5 μL of the amplified product along with 1.5 μL of a 1000 bp molecular weight marker. The electrophoretic run was performed at 100 V for 30 minutes, and the visualization of the fragments was done under UV light after staining with ethidium bromide.

#### 2.5.3 Sequencing

The amplified samples were sequenced using the Sanger method at CoreGenomics (USA). The obtained sequences were manually assembled using Sequencher v5.4.6 (Gene Codes, Ann Arbor, MI, USA), and subsequently aligned using MEGA X (46). The curated sequences were initially compared with available records in GenBank through BLAST searches in the NCBI database, and later with records from BOLD Systems to confirm the taxonomic identity.

#### 2.5.4 Phylogenetic analysis

Phylogenetic inference was performed using the Maximum Likelihood method, employing RAxML-HPC BlackBox v8.2.12, with 1000 bootstrap replicates, available on the CIPRES Science Gateway platform. The resulting phylogenetic tree was visualized and edited in FigTree v1.4.4, adjusting the orientation of the clades, substitution scale, and nodal support values for proper interpretation. The sequences obtained were deposited in the NCBI GenBank under the accession codes (PZ005867-PZ005885).

#### 2.5.5 Genetics distances

Genetic distances were calculated under the Kimura 2-Parameter (K2P) model in MEGA X for *Anastrepha* spp. Intraspecific divergence was estimated as the mean genetic distance within each species (Mean % Intra-Sp), whereas interspecific divergence was determined based on the minimum genetic distance to the nearest neighbor (Nearest Neighbor, NN) and the maximum genetic distance observed among species.

## 3 Results

To identify fruit flies morphologically and molecularly, traps were installed in fruit orchards in the province of Luya. This confirmed their presence and supported the collection of infested fruits and the subsequent rearing of specimens under controlled laboratory conditions. This procedure resulted in the emergence of 3688 adult flies. Analysis of the sex ratio revealed a significant deviation from the expected 1:1 distribution (χ² = 52.02, df = 1, p < 0.001). Of the total individuals that emerged, 55.9% were females (95% confidence interval: 0.543–0.575), while 44.1% were males. This corresponds to a sex ratio of 1.3 females per male, demonstrating that the proportion of females was significantly higher than that of males. Only adult females were used for morphological identification, and these same specimens were employed in the molecular analyses. After confirming taxonomic identity using both approaches, we evaluated species preference for specific fruit hosts.

### 3.1 Morphological identification

**Figure 2:**
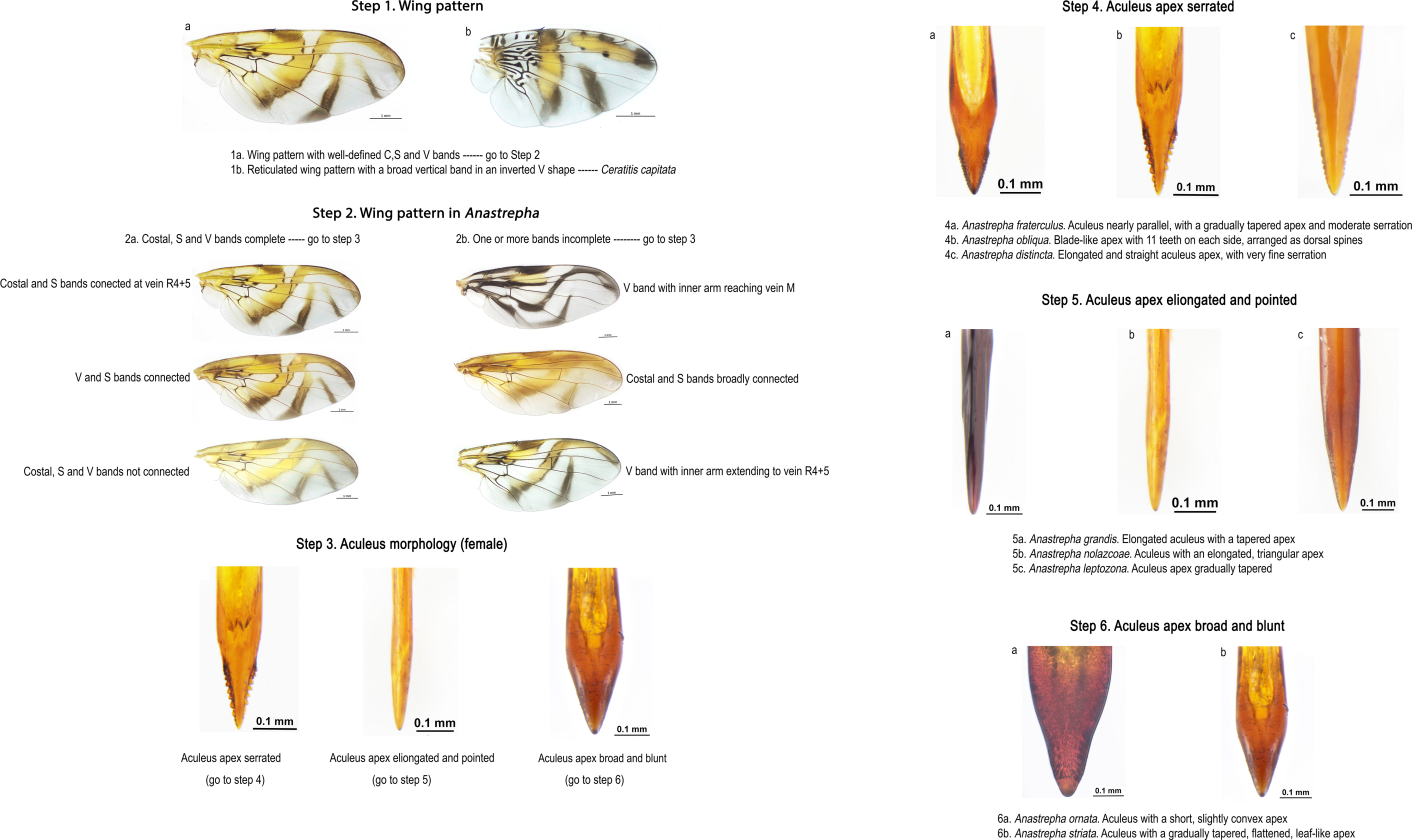
Pictorial keys for the morphological identification of fruit fly species, based on wing patterns and aculeus morphology in females. The differentiation of the wing pattern of the *Anastrepha* and *Ceratitis* genera is presented. Additionally, the classification of the wing pattern of the *Anastrepha* genus is described, based on the characteristics of the C, S, and V bands, classified as complete or incomplete. Three distinct types of aculeus tips are also illustrated: serrated, long and fine, and wide, which serve as key characteristics for differentiating the identified species.

**Figure 3:**
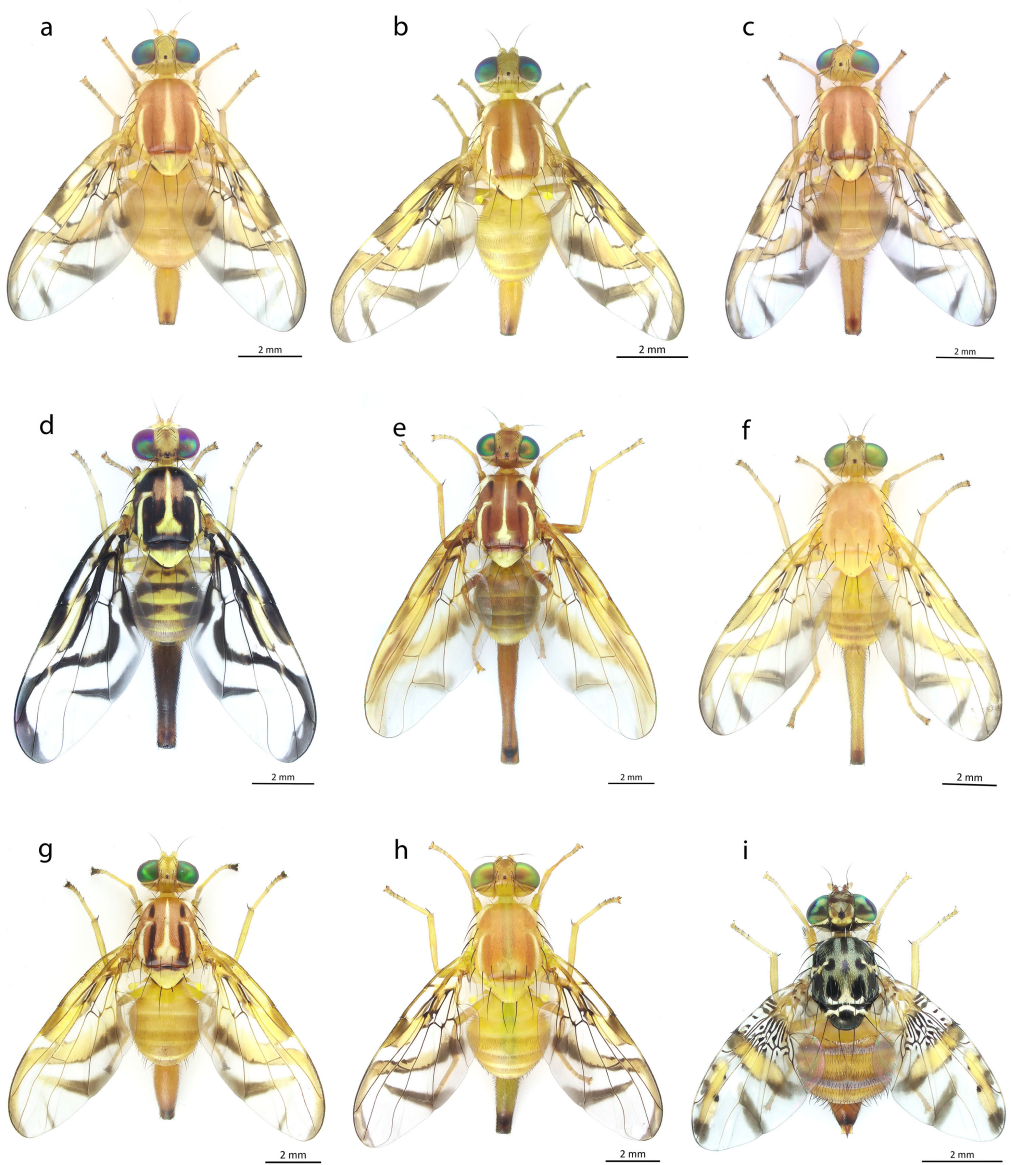
Adult females of the identified fruit fly species: **(a)** *Anastrepha fraterculus*, **(b)** *Anastrepha obliqua*, **(c)** *Anastrepha distincta*, **(d)** *Anastrepha ornata*, **(e)** *Anastrepha grandis*, **(f)** *Anastrepha nolazcoae*, **(g)** *Anastrepha striata*, **(h)** *Anastrepha leptozona* and **(i)** *Ceratitis capitata*.

For morphological identification, pictorial keys were created based on the wing pattern and the shape of the aculeus tip of adult females (**Figure 2**). In step 1, the genera *Anastrepha* and *Ceratitis* were distinguished using the wing patterns. In step 2, the wing pattern of the *Anastrepha* genus was classified based on the presence or absence of the C, S, and V bands, complete or incomplete. In step 3, three types of aculeus tips were differentiated: serrated, long and fine, and wide. Finally, in steps 4, 5, and 6, the species associated with each type of aculeus described in step 3 were identified. The identified species were: *Anastrepha fraterculus*, *Anastrepha. obliqua*, *Anastrepha distincta*, *Anastrepha ornata*, *Anastrepha grandis*, *Anastrepha nolazcoae*, *Anastrepha striata*, *Anastrepha leptozona*, and *Ceratitis capitata* (genus *Ceratitis*), all of which are illustrated in **Figure 3**.

### 3.2 Molecular identification

**Figure 4:**
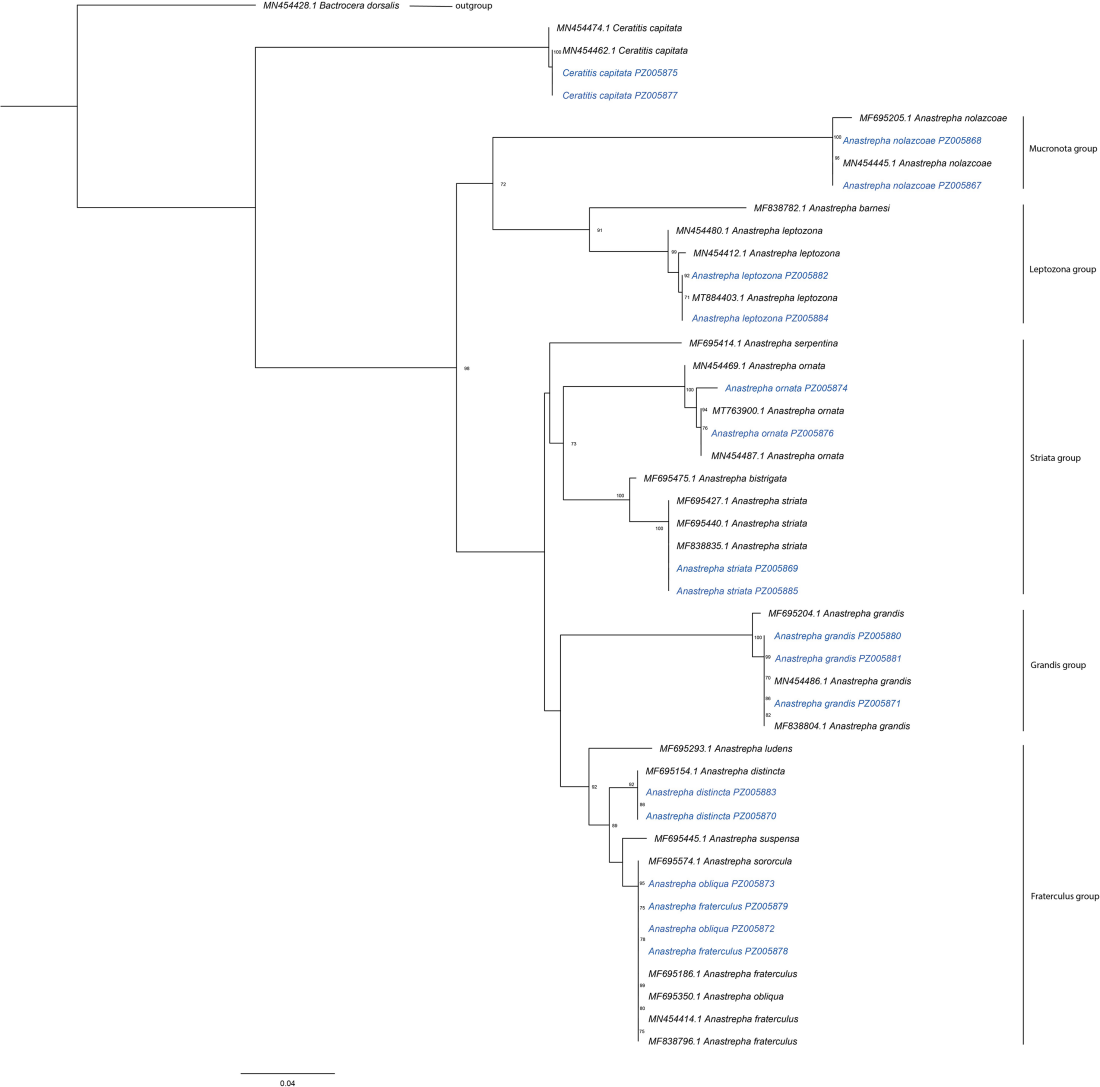
Phylogenetic tree inferred using Maximum Likelihood (RAxML) from mitochondrial cytochrome c oxidase subunit I (Cox1) gene sequences. The reference sequences used are shown in black, while the sequences obtained in this study are represented in blue. The tree displays the clade structure corresponding to the main species groups of the genus *Anastrepha* (*fraterculus, striata, grandis, leptozona, and mucronot*a), as well as a clearly differentiated clade for *Ceratitis capitata*. The species used as the outgroup is explicitly indicated.

The molecular analysis based on the mitochondrial cytochrome c oxidase subunit I (cox 1) marker reliably identified 7 species of *Tephritidae*. In the phylogenetic tree (**Figure 4**), inferred using Maximum Likelihood (RAxML) with 1000 bootstrap replicates, the sequences obtained in this study (represented in blue) grouped consistently with the reference sequences (in black), which are detailed with GenBank codes and BINs from the BOLD system (**Table 2**).

**Table 2:** Records of mitochondrial cytochrome oxidase subunit I (cox1) gene sequences used in the molecular analysis of fruit fly species, indicating GenBank accession numbers, Barcode Index Numbers (BIN), and country of origin.

Species	Genbank	BIN	Country
*Anastrepha fraterculus*	MF695186	BOLD: AAC0699	Peru
	MF838796	BOLD: AAC0699	Peru
	MN454414	BOLD: AAC0699	Peru
*Anastrepha obliqua*	MF695350	BOLD: AAC0699	Bolivia
*Anastrepha distincta*	MF695154	BOLD: AAC0699	Panamá
*Anastrepha ornata*	MN454487	BOLD: ADH6476	Peru
	MN454469	BOLD: ADH6476	Peru
	MT763900	BOLD: ADH6476	Ecuador
*Anastrepha grandis*	MF695204	BOLD: AAE5912	Colombia
	MF838804	BOLD: AAE5912	Bolivia
	MN454486	BOLD: AAE5912	Peru
*Anastrepha nolazcoae*	MF695205	BOLD: ACI4134	Peru
	MT884396	BOLD: ACI4134	Ecuador
	MN454445	BOLD: ACI4134	Peru
*Anastrepha striata*	MF695440	BOLD: AAC9702	Mexico
	MF695427	BOLD: AAC9702	Peru
	MF838835	BOLD: AAC9702	El Salvador
*Anastrepha leptozona*	MN454480	BOLD: AAR3707	Peru
	MN454412	BOLD: AAR3707	Peru
	MT884403	BOLD: AAR3707	Ecuador
*Ceratitis capitata*	MN454474	BOLD: AAA3297	Peru
	MN454462	BOLD: AAA3297	Peru
*Anastrepha ludens*	MF695293	BOLD: AAJ2068	Guatemala
*Anastrepha serpentina*	MF695414	BOLD: AAF3739	Peru
*Anastrepha suspensa*	MF695445	BOLD: AAC0699	Puerto Rico
*Anastrepha sororcula*	MF695574	BOLD: AAC0699	Brasil
*Anastrepha bistrigata*	MF695475	BOLD: AAC9702	Brasil
*Anastrepha barnesi*	MF838782	BOLD: AAR3728	Peru
*Bactrocera dorsalis*	MN454428	BOLD: AAA2295	South Africa

The presence of *Anastrepha distincta* (*fraterculus* group), *A. ornata* and *A. striata* (*striata* group), *A. grandis* (*grandis* group), *A. leptozona* (*leptozona* group), *A. nolazcoae* (*mucronota* group), and *Ceratitis capitata* was confirmed, with their sequences forming well-defined clades congruent with the reference records, with bootstrap support values ≥ 70%. In contrast, the COI marker did not allow clear differentiation between *Anastrepha fraterculus, Anastrepha sororcula*, and *Anastrepha obliqua*, all included in the *fraterculus* group, as the corresponding sequences grouped in the same clade, without evident genetic separation (**Figure 4**).

### 3.3 Genetics distances

The mean intraspecific divergence ranged from 0.00% to 0.70% (**Table 3**). The lowest values (0.00%) were recorded in *Anastrepha fraterculus*, *A. obliqua*, and *A. striata*, whereas the highest value corresponded to *A. ornata* (0.70%). Intermediate values were observed in *A. grandis* (0.28%), *A. nolazcoae* (0.43%), *A. distincta* (0.45%), and *A. leptozona* (0.52%).

**Table 3:** Intraspecific and interspecific genetic distances of *Anastrepha* species in our study, and their nearest neighbors (NN), calculated using the Kimura 2-parameter model.

Species	Min % Inter-sp (Distance to NN)	Max % Inter-sp	Mean % Intra-sp	Nearest neighbor (NN)
*Anastrepha fraterculus*	0	13.5	0	*Anastrepha obliqua*
*Anastrepha obliqua*	0	13.4	0	*Anastrepha fraterculus*
*Anastrepha distincta*	1.7	13.7	0.5	*Anastrepha sororcula*
*Anastrepha ornata*	7.4	13.7	0.7	*Anastrepha sororcula*
*Anastrepha grandis*	9.7	16.7	0.3	*Anastrepha fraterculus*
*Anastrepha nolazcoae*	13.6	16.4	0.4	*Anastrepha sororcula*
*Anastrepha striata*	2.2	15.1	0	*Anastrepha bistrigata*
*Anastrepha leptozona*	8.4	15.1	0.5	*Anastrepha barnesi*

The minimum interspecific distances ranged from 0.00% to 13.56%. The lowest divergence was observed between *A. fraterculus* and *A. obliqua* (0.00%), indicating no detectable nucleotide differences in the analyzed COI fragment, with both species constituting reciprocal nearest neighbors. *A. distincta* showed a divergence of 1.69% relative to *A. sororcula*; *A. striata*, 2.15% relative to *A. bistrigata*; *A. ornata*, 7.40% relative to *A. sororcula*; *A. leptozona*, 8.37% relative to *A. barnesi*; *A. grandis*, 9.71% relative to *A. fraterculus*; and *A. nolazcoae*, 13.56% relative to *A. sororcula*. Maximum interspecific distances reached values of up to 16.66%, with 13.55% in *A. fraterculus*, 13.42% in *A. obliqua*, 13.68% in *A. distincta*, 13.66% in *A. ornata*, 16.66% in *A. grandis*, 16.42% in *A. nolazcoae*, 15.06% in *A. striata*, and 15.08% in *A. leptozona*.

### 3.4 Species richness and abundance by host plant

A stacked bar chart was created to assess whether fruit flies show a preference for any of the 19 fruit species (**Supplementary Table 2**). The chart shows the number of adult fruit flies of each species associated with each fruit species.

**Figure 5:**
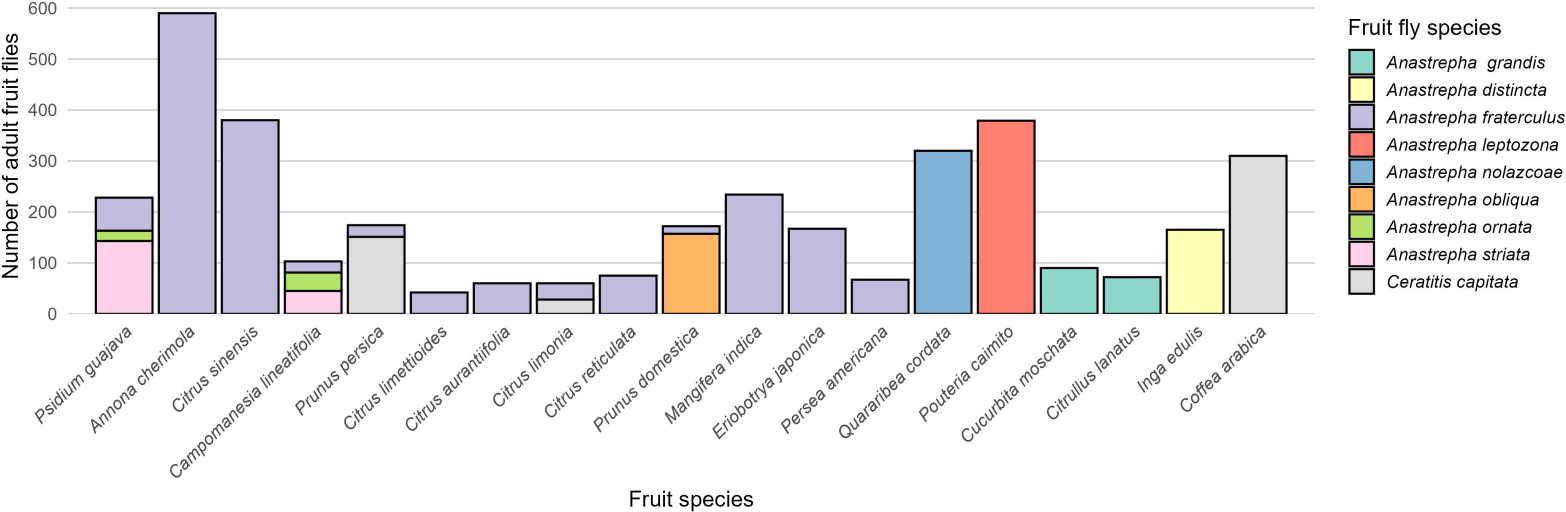
Adult fruit fly abundance by species and fruit host type in the province of Luya, Amazonas region.

The maximum and minimum species richness of fruit flies associated with a single fruit species were three and one, respectively. The highest abundance of adults was recorded in guava (*P. guajava*) with 143 A*. striata*, 20 A*. ornata*, and 65 A*. fraterculus*, as well as in palillo (*C. lineatifolia*) with 45, 36, and 22 individuals of these species, respectively. *A. fraterculus* exhibited the greatest host range, being associated with a total of 13 fruit species. In cherimoya (*A. cherimola*), A. fraterculus was the only species recorded, and this host yielded the highest number of emerged adults (590 individuals). In contrast, *C. capitata* infested peach (*P. persica*), mandarin (*C. limonia*), and coffee (*C. arabica*), yielding 151, 28, and 310 individuals, respectively (**Figure 5**).

## 4 Discussion

In the province of Luya, a wide variety of native and introduced fruit trees are cultivated, primarily in family orchards (**Supplementary Table 3**). These orchards are an important source of income for local families, and the fruit is sold in local markets. These orchards are important for household food security because they provide income and food for consumption (47). The diversity and continuity of host plants in the province of Luya could favor the persistence of Tephritidae populations by acting as a refuge against fruit fly eradication programs implemented in nearby regions such as Lambayeque and La Libertad.

In the present study, the laboratory rearing of fruit flies showed a sex ratio of 1.3:1 (females:males). This result is consistent with that reported by Nolasco and Iannacone (48), who found a ratio of 1.7:1 (females:males) following capture with traps in fruit plantations. The higher proportion of females could be due to differential survival during the larval and pupal stages, favored by the conditions established in the laboratory. According to Fisher’s principle, the expected sex ratio is 1:1, but differential mortality and competition during pupation can produce a bias in the adult stage (49). Furthermore, Nolasco and Iannacone (48) suggest that males seek to mate repeatedly while females prioritize feeding and then oviposition, which could explain the higher capture of females in food-baited traps. These findings suggest that both differential mortality at early stages and adult feeding and reproductive behavior contribute to a higher proportion of females than males.

Morphological identification reliably discriminated the nine Tephritidae species recorded, using key diagnostic characters such as the wing pattern, thorax morphology, and aculeus shape, which are widely recognized as fundamental in the taxonomy of the group (38). However, in taxonomic complexes like the *Anastrepha fraterculus* complex, the overlap of morphological traits may restrict identification based solely on morphology, which aligns with the findings of Barr et al. (50) and underscores the need to integrate molecular tools for a more robust species delimitation.

Integrative taxonomy is a more robust approach that combines genetic, morphological, and ecological evidence (51, 52). It is particularly relevant in species complexes with high interspecific similarity and has proven crucial for distinguishing closely related species. For example, it was instrumental in defining the *fraterculus* group (53). The combined use of molecular methods and morphological identification optimizes accurate species delimitation in insects, especially in species complexes or among morphologically similar taxa (54, 55).

Molecular analysis based on the mitochondrial cytochrome c oxidase subunit I (COI) marker effectively supported the identification of most recorded Tephritidae species. However, it was limited in discriminating between *Anastrepha fraterculus* and *A. obliqua*. Barr et al. (34) previously reported this pattern when they demonstrated that COI-based DNA barcoding cannot reliably distinguish between these two species. Similarly, Bartolini et al. (56) found that several species within the *fraterculus* group share the same barcode index (BIN) when using this marker, which highlights its limited resolving power within this species complex.

The intraspecific genetic divergence observed in this study was low, ranging from 0.00% to 0.70%. *Anastrepha fraterculus*, *A. obliqua*, and *A. striata* exhibited the lowest values, suggesting high genetic homogeneity within these species’ populations, while *A. ornata* showed the highest divergence. These values are lower than those reported for *Anastrepha* spp. by Barr et al. (34), who found an intraspecific variation range of 0.5% to 3.32%, and by Bartolini et al. (56), who found values ranging from 1.01% to 3%.

The interspecific divergence among the evaluated species ranged from 0% to 16.7%. The lowest divergence, 0%, was observed between *A. fraterculus* and *A. obliqua*. According to Barr et al. (34), this indicates that these *fraterculus* group species share the same haplotypes. In contrast, the highest values were recorded between *A. grandis* and *A. nolazcoae* (16.7%), reflecting greater genetic differentiation between these species. Our results align with Bartolini et al. (56) findings that *A. nolazcoae* and *A. grandis* exhibited the greatest interspecific distances, ranging from 16.25% to 17.12%.

In this context, recent studies have proposed using complementary molecular markers to improve taxonomic resolution within the f*raterculus* group. Gomulski et al. (57) suggest that the nuclear ITS2 marker enables more precise discrimination among morphologically similar species and that combining it with protein-coding genes, such as EF1α, increases the robustness of phylogenetic analyses. Additionally, analyzing ribosomal internal transcribed spacer 1 (ITS1) fragments has been shown to be an effective method for evaluating genetic variation within the *A. fraterculus* complex (58–60).

The morphological and molecular identification of the nine fruit fly species allowed each species to be associated with the fruits from which they emerged, which facilitated the evaluation of host preference of the flies in the province of Luya. The highest abundance of fruit flies was found in guava and palillo, both belonging to the same family, Myrtaceae, where the same three species were recorded. The nutritional composition of the fruits influences, at least in part, their suitability as hosts for the larvae of fruit flies, since it provides the nutrients necessary for their development and survival (61, 62). On the other hand, the preference of *A. fraterculus* for a wide number of fruit species was also reported by Coelho et al. (63), who found it infesting 20 hosts in five botanical families, including the species *Citrus sinensis*, *Psidium guajava*, *Prunus persica*, and *Citrus limonia*, which also coincided as hosts of *A. fraterculus* in our study, which could be due to its wide distribution in the Neotropical region, its high ecological plasticity, and its highly polyphagous nature (17). Likewise, the high number of flies emerging from cherimoya is explained by the tendency of females to lay more eggs in fruits that offer greater chances of survival for their offspring (64). Likewise, an exclusive association of *Anastrepha grandis* with hosts of the family Cucurbitaceae was observed, reflecting a marked host preference and a high degree of specialization, in addition to agreeing with experimental evidence showing higher infestation rates and larval survival of this species in fruits of *Cucurbita* spp. and *Citrullus* spp. (65). Finally, the record of *C. capitata*, an exotic species with high phytosanitary impact, confirms its establishment in the region and its expansion at the national level. Castillo-Nole and Ortiz-Arzapalo (66) monitored *C. capitata* in *Passiflora ligularis*, *C. arabica*, and citrus crops in the Pasco region, Peru, which supports its presence and expansion in different areas of the country.

## 5 Conclusions

Using morphological and molecular tools, nine species of fruit fly associated with 19 fruit species were identified for the first time in the province of Luya, Amazonas region. A preference for *Psidium guajava* and *Campomanesia lineatifolia* was observed, and polyphagy was recorded in *Anastrepha fraterculus* and *Ceratitis capitata.*

## Data Availability

The original contributions presented in the study are publicly available. This data can be found here: GitHub, https://github.com/WAHGNERM2002/FRUIT-FLY; NCBI GenBank, accessions PZ005867–PZ005885.
