# Supplementary material for: Fruit fly species (Diptera: Tephritidae) associated with fruit orchards in the province of Luya, Amazonas, Peru
Source: Front Insect Sci. 2026 Mar 18;6:1789891. doi: 10.3389/finsc.2026.1789891 (PMC13038917; doi:10.3389/finsc.2026.1789891)
Supplement: Supplementary file 1 [file DataSheet1.pdf]

**Publication**	**Fruit fly species (Diptera: Tephritidae) associated with fruit orchards in the northeastern region of Peru**
**Supplementary Table 1**	Geographic location of installed traps and associated host plants for fruit fly monitoring in Luya Province, Amazonas, Peru.
**Supplementary Table 2**	Distribution of adult fruit fly specimens (genera Anastrepha and Ceratitis) by species and fruit host type
**Supplementary Table 3**	Fruit fly species (Diptera: Tephritidae) associated with fruit orchards in the province of Luya, Amazonas, Peru, and their host species, according to native or introduced origin.
**Supplementary Table 4**	Author contributions according to the CRediT (Contributor Roles Taxonomy) classification.

Git hub: https://github.com/WAHGNERM2002/FRUIT-FLY

STable1

N° TRAPS	HOST	LOCATION	LATITUDE	LONGITUDE
1	Guayaba	Playa de Jumeth	6°15'58.67"S	78°16'28.44"O
2	Guaba	Tactamal	6°15'26.73"S	78°13'45.76"O
3	Mango	Motupe	6°16'56.51"S	78°13'5.55"O
4	Limon mandarina	Motupe	6°17'2.67"S	78°13'1.44"O
5	Cafe	Tactamal	6°15'43.28"S	78°12'48.65"O
6	Ciruelo	San Juan de Ocumal	6°16'45.91"S	78° 9'57.76"O
7	Naranja	Tactamal	6°17'39.21"S	77°58'26.11"O
8	Chirimoya	Ribera del Utcubamba	6°36'3.53"S	77°48'54.33"O
9	Guayaba	Congon	6°18'37.36"S	78° 6'23.50"O
10	Naranja	Lonya Chico	6°13'47.55"S	77°57'18.36"O
11	Chirimoya	Palto	6°12'12.57"S	78°18'0.29"O
12	Nispero	San Miguel de Poro Poro	6°20'16.45"S	78°11'15.27"O
13	Palillo	Carmelo	6°17'28.84"S	78°15'27.91"O
14	Naranja	Ribera del Utcubamba	5°59'39.77"S	77°59'12.01"O
15	Lima	Ribera del Utcubamba	6°39'58.24"S	77°48'3.12"O

16	Limon dulce	Ribera del Utcubamba	6° 1'57.91"S	77°56'34.35"O
17	Zapote	Ribera del Utcubamba	6° 0'28.41"S	77°57'32.29"O

Stable2

FRUIT	***Anastrepha fraterculus***	***Anastrepha striata***	***Anastrepha ornata***	***Anastrepha nolazcoae***	***Anastrepha leptozona***	***Anastrepha grandis***	***Anastrepha obliqua***	***Anastrepha distincta***	***Ceratitis capitata***
Psidium guajava	65	143	20	0	0	0	0	0	0
Annona cherimola	590	0	0	0	0	0	0	0	0
Citrus sinensis	380	0	0	0	0	0	0	0	0
Campomanesia lineatifolia	22	45	36	0	0	0	0	0	0
Prunus persica	23	0	0	0	0	0	0	0	151
Citrus limettioides	42	0	0	0	0	0	0	0	0
Citrus aurantiifolia	60	0	0	0	0	0	0	0	0
Citrus limonia	32	0	0	0	0	0	0	0	28
Citrus reticulata	75	0	0	0	0	0	0	0	0
Prunus domestica	15	0	0	0	0	0	157	0	0
Mangifera indica	234	0	0	0	0	0	0	0	0
Eriobotrya japonica	167	0	0	0	0	0	0	0	0
Persea americana	67	0	0	0	0	0	0	0	0
Quararibea cordata	0	0	0	320	0	0	0	0	0

Pouteria caimito	0	0	0	0	379	0	0	0	0
Cucurbita moschata	0	0	0	0	0	90	0	0	0
Citrullus lanatus	0	0	0	0	0	72	0	0	0
Inga edulis	0	0	0	0	0	0	0	165	0
Coffea arabica	0	0	0	0	0	0	0	0	310

Stable3

Species of fruit fly	Host			
	**Common name**	**Scientific name**	**Family**	**Type**
	Orange	*Citrus sinensis*	Rutaceae	Introduced
	Mandarin	*Citrus reticulata*	Rutaceae	Introduced
	Lime	*Citrus aurantiifolia*	Rutaceae	Introduced
	Sweet lime	*Citrus limettioides*	Rutaceae	Introduced
	Mandarin lime	*Citrus limonia*	Rutaceae	Introduced
	Loquat	*Eriobotrya japonica*	Rosaceae	Introduced
	Plum	*Prunus domestica*	Rosaceae	Introduced
	Peach	*Prunus persica*	Rosaceae	Introduced
	Guava	*Psidium guajava*	Myrtaceae	Native
	Palillo	*Campomanesia lineatifolia*	Myrtaceae	Native
	Mango	*Mangifera indica*	Anacardiaceae	Introduced
	Avocado	*Persea americana*	Lauraceae	Introduced
Anastrepha fraterculus	Cherimoya	*Annona cherimola*	Anonnaceae	Native
Anastrepha striata y Anastrepha ornaa	Palillo	*Campomanesia lineatifolia*	Myrtaceae	Native

	Guava	*Psidium guajava*	Myrtaceae	Native
	Chiclayo	*Cucurbita moschata*	Cucurbitaceae	Native
Anastrepha grandis	Watermelon	*Citrullus lanatus*	Cucurbitaceae	Native
Anastrepha distincta	Ice-cream bean	*Inga edulis*	Fabaceae	Native
Anastrepha obliqua	Plum	*Prunus domestica*	Rosaceae	Native
Anastrepha leptozona	Star apple	*Pouteria caimito*	Sapotaceae	Introduced
Anastrepha nolazcoae	Sapote	*Quararibea cordata*	Malvaceae	Introduced
	Peach	*Prunus persica*	Rosaceae	Introduced
	Coffee	*Coffea arabica*	Rutaceae	Introduced
Ceratitis capitata	Mandarine lime	*Citrus limonia*	Rubiaceae	Introduced
